# A new formulation of Gamma Delta Tocotrienol has superior bioavailability compared to existing Tocotrienol-Rich Fraction in healthy human subjects

**DOI:** 10.1038/srep13550

**Published:** 2015-09-01

**Authors:** Puvaneswari Meganathan, Rafid Salim Jabir, Ho Gwo Fuang, Nirmala Bhoo-Pathy, Roma Basu Choudhury, Nur Aishah Taib, Kalanithi Nesaretnam, Zamri Chik

**Affiliations:** 1 Department of Surgery, Faculty of Medicine, University of Malaya, 50603 Kuala Lumpur, Malaysia; 2 Oncology Clinical Unit, Faculty of Medicine, University of Malaya, 50603 Kuala Lumpur, Malaysia; 3 Department of Social and Preventive Medicine (SPM), Faculty of Medicine, University of Malaya, 50603 Kuala Lumpur, Malaysia; 4 Department of Pharmacology, Faculty of Medicine, University of Malaya, 50603 Kuala Lumpur, Malaysia; 5 Malaysian Palm Oil Board (MPOB), No. 6 Persiaran Institusi, Bandar Baru Bangi, 43000 Kajang, Selangor, Malaysia; 6 Clinical Investigation Centre, Faculty of Medicine, University of Malaya, 50603 Kuala Lumpur, Malaysia

## Abstract

Gamma and delta tocotrienols are isomers of Vitamin E with established potency in pre-clinical anti-cancer research. This single-dose, randomized, crossover study aimed to compare the safety and bioavailability of a new formulation of Gamma Delta Tocotrienol (GDT) in comparison with the existing Tocotrienol-rich Fraction (TRF) in terms of gamma and delta isomers in healthy volunteers. Subjects were given either two 300 mg GDT (450 mg γ-T3 and 150 mg δ-T3) capsules or four 200 mg TRF (451.2 mg γ-T3 & 102.72 mg δ-T3) capsules and blood samples were taken at several time points over 24 hours. Plasma tocotrienol concentrations were determined using HPLC method. The 90% CI for gamma and delta tocotrienols for the ratio of log-transformation of GDT/TRF for C_max_ and AUC_0–∞_ (values were anti-logged and expressed as a percentage) were beyond the bioequivalence limits (106.21–195.46, 154.11–195.93 and 52.35–99.66, 74.82–89.44 respectively). The Wilcoxon Signed Rank Test for T_max_ did not show any significant difference between GDT and TRF for both isomers (p > 0.05). No adverse events were reported during the entire period of study. GDT was found not bioequivalent to TRF, in terms of AUC and C_max_. Gamma tocotrienol in GDT showed superior bioavailability whilst delta tocotrienol showed less bioavailability compared to TRF.

Vitamin E is found in abundance in vegetable oils with tocopherols as the main constituent. In the past, tocopherols have been vastly studied where else there is still paucity in clinical data where tocotrienols are concerned. Palm oil contains about 70% tocotrienols of the vitamin E content; making it one of the richest source of natural tocotrienols. Both tocopherols and tocotrienols have similar structures with the presence of a chromanol ring and a side chain positioned at C-2. Depending on the position and degree of methylation on the chromanol head, tocopherols and tocotrienols can be further differentiated into 4 isomers of α, β, γ and δ respectively. However, tocotrienols possess unsaturated isoprenoid side chain with 3 double bonds at carbon 3, 7 and 11 unlike tocopherols with saturated phytyl chain. This unsaturated side chain of tocotrienols facilitates its efficient penetration into tissues with major composition of fatty layers such as adipose, liver and brain^1^,^2^,^3^. Research have proven that tocotrienols demonstrate various biological and physiological properties such as cardioprotective^4^, neuroprotective^5^, anti-cancer activity^6^ and cholesterol-lowering effects^7^, therefore distinguishing them from tocopherols.

Bioavailability is defined as the rate and extent of the absorption and availability of the active constituent or its moiety at the target site^8^. Alpha-tocopherol is the most abundant form of Vitamin E detected in circulating plasma. Meanwhile, the bioavailability of tocotrienols in plasma has been reported at a much lower concentration than alpha-tocopherol^9^,^10^. The absolute bioavailability of alpha tocotrienol was reported as 28% followed by gamma and delta tocotrienols at only about 9%^11^. As a result, self-emulsifying systems were established to achieve higher absorption for tocotrienols^12^. Tocotrienol-rich Fraction (TRF) [Tocovid Suprabio^TM^], is an over-the counter vitamin E supplement, containing alpha, gamma and delta tocotrienols in addition to α-tocopherol, phytosterol, phytocarotenoid complex and plant squalene.

Several anti-cancer studies indicated that gamma and delta tocotrienols exhibited more potent anti-cancer activity than other isomers of tocotrienols and alpha-tocopherol^13^. In pre-clinical studies, the IC_50_ concentration of gamma tocotrienol required to induce apoptosis in both MDA-MB-231 and MCF-7 human breast cancer cell lines was remarkably lower than delta tocotrienol^14^. Similar observation was also demonstrated in a study by Guthrie *et al*.^15^ (1998) using MCF-7 cell line, wherein the IC_50_ concentration of gamma tocotrienol was only 30 μg/mL as compared to 90 μg/mL of delta tocotrienol indicating increased anti-cancer activity.

Hence, a new formulation of Gamma Delta Tocotrienol (GDT) which contains only gamma and delta isomers (75:25) had been developed in order to improve the effectiveness and enhance the delivery of the gamma and delta isomers as compared to the currently available TRF formulation. Therefore, in this study, we aimed to evaluate the pharmacokinetics and bioavailability of a new formulation of Naturale^3^ Gamma Delta Tocotrienol (GDT) with the available Tocotrienol-rich fraction (TRF) in healthy volunteers.

## Subjects and Methods

### Study design and medications

This was an open label, randomized, 2-way cross-over study with 2 treatments and 2 periods with one week wash-out period. The study protocol and informed consent forms were reviewed and approved by the medical ethics committee of University Malaya Medical Centre (UMMC) (protocol no:944.62). This study was registered at clinicaltrial.gov with the Identifier NCT01571921 on 26 March 2012. Volunteers were provided with both verbal and written information on the nature of study including the aim of the study, methods to be employed and possible risks related to the drugs. Written consent was obtained prior to the initiation of screening and trial procedures.

The trial protocol was in compliance with Declaration of Helsinki^16^, Malaysian Guidelines for Good Clinical Practice^17^ and Malaysian Guidelines for the Conduct of Bioavailability and Bioequivalence Studies^18^. The volunteers were randomized to receive one of the study formulations. The test formulation, Naturale^3^ Gamma delta tocotrienol (GDT) (consist of 2 capsules of 300 mg each containing 450 mg γ-T3 and 150 mg δ-T3) was provided by Davos Life Science Pte Ltd, Singapore. The reference drug is the commercially available Tocovid Suprabio^TM^ which comprises of 4 capsules of 200 mg containing 451.2 mg γ-T3 and 102.72 mg δ-T3 obtained over the shelf.

### Subjects

Healthy volunteers were recruited through advertisement and medical screening were conducted to exclude any chronic illness or abnormalities, consumption of tobacco, alcohol, drugs and current or past use of any active substance. After a physical examination (to exclude any abnormality of the cardiovascular, respiratory, abdominal and central nervous system), blood pressure and pulse rate were measured and general examination (to rule out anemia, cyanosis, clubbing, jaundice and lymphadenopathy) of the subject was conducted to exclude any illness or abnormality. Resting blood pressure was recorded using a sphygmomanometer while the subject was in a sitting position. Blood sample was collected for full blood count, urea, electrolytes, liver and renal function tests, serology as well as random glucose test. All female volunteers were subjected to urinary pregnancy tests.

The recruitment was conducted based on reviews of pathology reports, medical history and in accordance to inclusion and exclusion criteria. At the end of study, blood sample was drawn again for assessment of all biochemical parameters for safety and tolerability. The subjects were then randomized into reference or test group. Blocked randomization method was used to generate the randomization list. Three blocks with block size of four was used. The drug containers were sequentially numbered and concealed until interventions were assigned.

### Inclusion and exclusion criteria

The healthy volunteers were recruited based on their age ranging from 21–55 years, in good health based on physical examination and pathological results. They are also literate, provided consent to the study protocols and do not have any reported allergies to vitamin E or palm oil.

The exclusion criteria comprises of pregnant or lactating mothers, chronic illness such as cardiovascular disease or hypertension, present or past history of cancer, history of bleeding tendencies with past or current use of antithrombotic drugs (aspirin or ticlopidine), anticoagulants (heparin, warfarin) and thrombolytic agents (streptokinase).

Those who are using investigational products or participated in any other clinical trials within 90 days prior to the initiation of trial were also excluded.

### Admission and study protocol

The subjects were admitted at Clinical examination ward of UMMC a day in advance to study (Day 0). After signed informed consent was obtained from all subjects, physical examination and general health status including body temperature, heart rate and blood pressure were monitored by a medical physician. A standardized meal was provided and subjects were required to fast from 10 p.m onwards. On day 1, at 7 a.m, an in-dwelling canula was placed in the antecubital vein of the subjects. A standardized breakfast which consists of 2 slices of white bread with chicken patty and warm water was provided followed by the dosing. 5 mL of blood was drawn into EDTA tubes before and at 0.5, 1, 2, 3, 4, 5, 6, 7, 8, 10, 14, and 24 hours after dosing. Standardized meals consisting of lunch, tea break and dinner were served at 4, 8 and 11 hours after dosing. Subjects were allowed to be involved in non-strenuous activities after dosing; however, they had to be in upright position for ±2 hours.

During the study, a medical physician who was blinded to the study drugs was present to monitor the general condition of subjects including vital signs such as overall well-being, blood pressure and heart rate as well as possible adverse effects of the drugs. All events either mild or serious were recorded on adverse effect forms. After 24 hours, subjects were discharged following the last blood sampling. Additional blood samples were drawn at the last time-point for blood profile and clinical chemistry tests for safety monitoring. After a one week wash out period, subjects returned to the ward and to be given the other formulation (test or reference) and the same procedures were repeated.

### Analysis of Plasma Samples

The plasma samples from this study were stored and analysed at Malaysian Palm Oil Board (MPOB). The HPLC method used to quantify concentrations of gamma and delta tocotrienol in plasma was validated as outlined in the USFDA Guidelines for Bioanalytical Analysis^19^. The parameters validated were specificity, linearity, accuracy, precision, recovery and stability.

The analysis of gamma and delta tocotrienols was performed using Agilent 1100 series HPLC System with *ChemStation Software* for LC System Rev. A.06.0x, *Instrument 2 Online* (Agilent Technologies, Malaysia) equipped with a quaternary pump maintained at a pressure of 24 ± 1 bar, a fluorescent detector and a degasser. An autosampler was set at the injection volume of 100 μL for determination of samples. Chromatographic separations were achieved using *Phenomenex®* Luna 5 u Silica 100 A ODS Hypersil column (250 × 4.60 mm i.d, 5 μm particle size; USA) operating at room temperature. The flow rate was set at 1 mL/min with excitation wavelength at 295 nm and emission wavelength at 325 nm. The total run time was 30 minutes. Isocratic elution was used and the mobile phase was prepared using 97.5% n-hexane, 2% Dioxane and 0.5% Isopropanol. The mobile phase was degassed by sonication prior to use. Liquid-liquid extraction method was used for plasma extraction. An internal standard, 2,2,5,7,8-Pentamethyl-6-hydroxychroman (PMC) was used.

**Figure 1:**
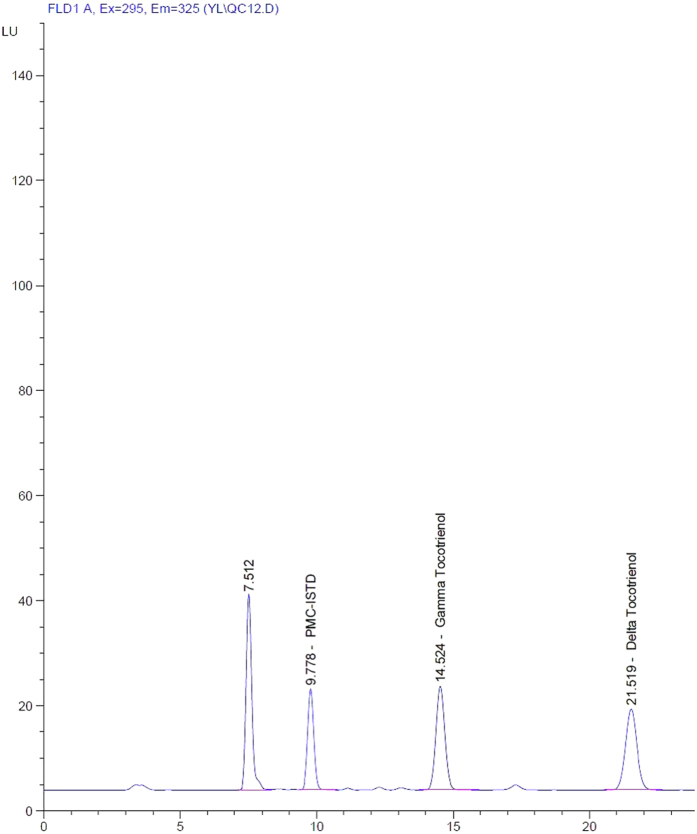
Chromatogram of gamma delta tocotrienols and internal standard in plasma.

**Figure 2:**
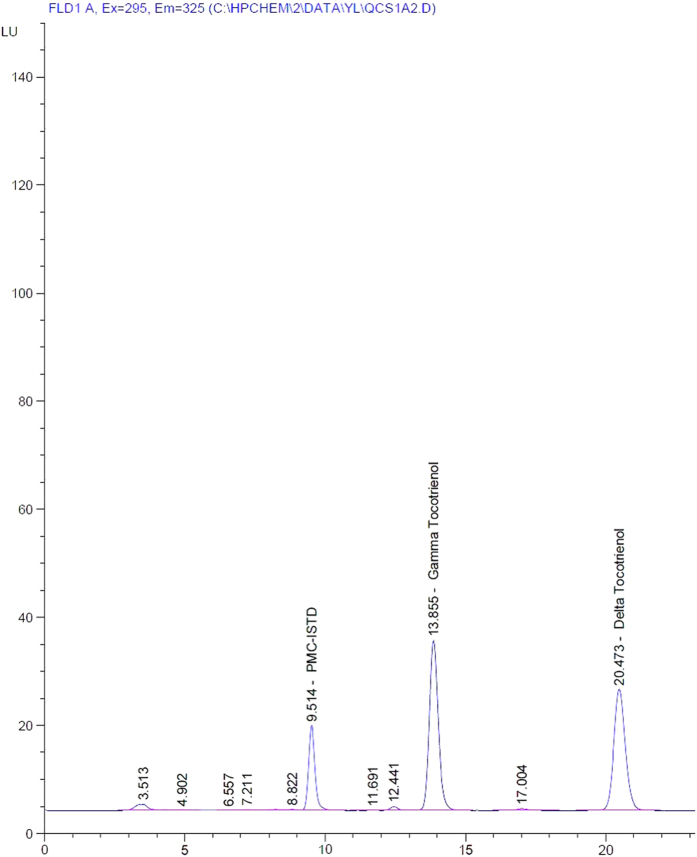
Chromatogram of gamma delta tocotrienols and internal standard in solvent.

The recovery of gamma tocotrienol ranged from 87.38% to 90.79% and it was between 81.39% to 90.33% for delta tocotrienol. Calibration curves of analytes spiked in plasma were linear with R^2^ > 0.990 from 1 μg/mL to 25 μg/mL. The concentrations of quality controls of gamma and delta tocotrienols used were 3 μg/mL, 13 μg/mL and 23 μg/mL as low, medium and high quality controls respectively. The coeffiecient of variation (CV) of intraday and interday ranged from 1.06 to 3.47 for gamma tocotrienol while it was from 0.66 to 5.19 for delta tocotrienol. Meanwhile, accuracy percentage values for gamma and delta tocotrienols ranged from 95.32% to 108.30% and 95.81% to 110.14% respectively. Both gamma and delta tocotrienols were also found to be stable after three freeze-thaw cycles. Figures 1 and 2 show chromatograms of gamma and delta tocotrienols prepared in plasma and solvent respectively.

### Pharmacokinetic analysis

Pharmacokinetic analysis was performed using non-compartmental analysis. All the pharmacokinetic parameters were calculated by using WinNonlin Professional version 5.3 (Pharsight Corporation, Mountain View, California). Peak plasma concentration (C_max_) and time to reach peak plasma concentration (T_max_) were obtained from the plasma concentration-time data. Area from time zero to last sampling time (AUC_0–24_) was determined using linear trapezoidal rule, while area from time zero to infinity (AUC_0–∞_) is the sum of AUC_0–24_ and AUC_t-∞_ (calculated by extrapolating plasma drug concentration (C_t_) to the time axis and dividing it with elimination rate constant (k_e_)). K_e_ was obtained from the slope of linear regression of the ln-transformed plasma concentration-time curve in the elimination phase. The elimination half-life (t_1/2_) was determined using the following equation 1:
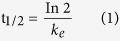


### Statistical Analysis

The sample size for this study was estimated using a power calculation conducted on the basis of data obtained from earlier tocotrienol studies^20^. Analysis of Variance (ANOVA) was used to obtain the significance of the bioavailability values for C_max_ and AUC_0–∞_. This was due to the variations that arise in cross-over studies in terms of subjects, formulations, sequences and periods. Analysis was conducted using WinNonlin Professional version 5.3. All the AUC and C_max_ values were adjusted accordingly based on the dose given before ANOVA was performed.

Bioequivalence testing was based on the 90% CI for the ratio of the population means (test formulation/reference formulation) for C_max_ and AUC^21^. The two formulations were considered to be bioequivalent only if the 90% CIs for the aforementioned values were within the predetermined bioequivalence range of 80%–125%. The AUC and C_max_ values were adjusted according to the dose given prior to ANOVA test. The difference in T_max_ values between the two formulations was assessed using nonparametric Wilcoxon signed rank test using SAS Enterprise guide 5.1.

## Results

### Demographic characteristics

Twelve healthy subjects (9 males, 3 females) were enrolled in this study. Their mean age and BMI were 24 ± 1.65 (range, 22–27) and 23.43 ± 3.5 (range, 18.87–28.2). Eleven out of twelve subjects completed the trial (Table 1). One subject dropped-out from the trial due to difficulties in obtaining blood samples and considering her safety and well-being, she was withdrawn from the study. The venipuncture site was normal and the subject was fine when she was allowed to go home.

**Figure 3:**
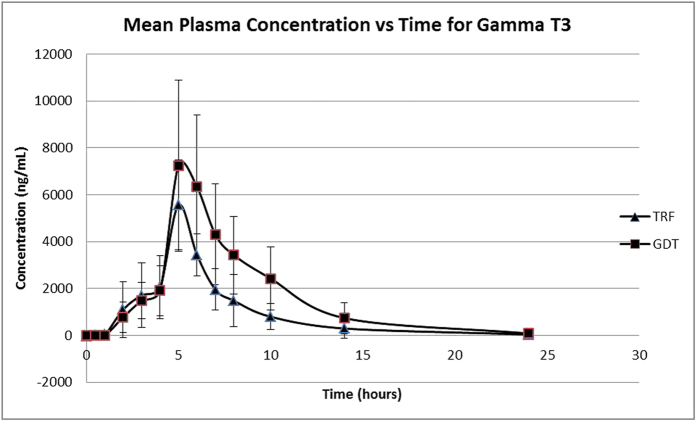
Mean plasma (SD) concentration of Gamma Tocotrienol versus time after single oral dose of 600 mg GDT and 800 mg TRF.

**Figure 4:**
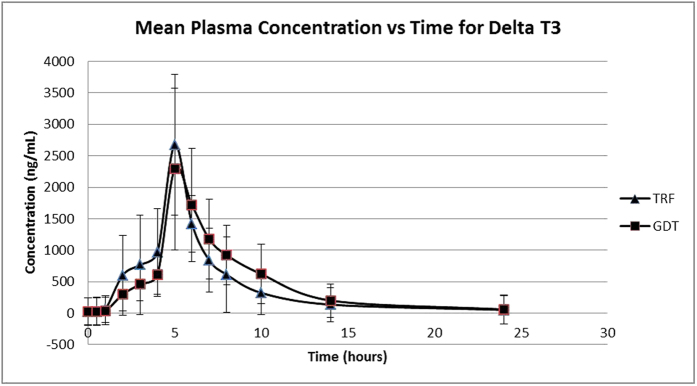
Mean plasma (SD) concentration of Delta Tocotrienol versus time after single oral dose of 600 mg GDT and 800 mg TRF.

The mean plasma concentration for gamma and delta tocotrienol versus time in the test and reference formulations is presented in Figures 3 and 4. The pharmacokinetic parameters are shown in Tables 2 and 3 for gamma and delta tocotrienol, respectively. The mean values for C_max_, T_max_, AUC_0–24_ and AUC_0–∞_ for gamma-tocotrienol in GDT were 8406.75 ± 3670.99 μg/L, 5.64 ± 1.50 hours, 39811.07 ± 13336.74 μg/L*h and 41091.37 ± 13406.89 μg/L*h respectively; while for TRF, the values were 5604.67 ± 1971.73 μg/L, 4.73 ± 0.90 hours, 23312.73 ± 9804.14 μg/L*h and 24256.29 ± 10591.03 μg/L*h.

Meanwhile, the mean values for C_max_, T_max_, AUC_0–24_ and AUC_0–∞_ for delta tocotrienol in GDT were 2693.89 ± 962.7 μg/L, 5.18 ± 0.40 hours, 12136.42 ± 6417.29 μg/L*h and 14627.95 ± 9792.93 μg/L*h respectively; while for TRF, the values were 2619.99 ± 1355.89 μg/L, 5.18 ± 1.83 hours, 10454.42 ± 6042.03 μg/L*h and 12453.83 ± 8875.65 μg/L*h respectively.

Tables 4 and 5 show the 90% CIs and the mean ratio of the test-to-reference formulations for C_max_ and AUC_0–∞_ for gamma and delta tocotrienols in the two different formulations. The bioequivalence analysis for CIs was based on the ratio of the population means (test formulation/reference formulation). These values were anti-logged and expressed as a percentage. The C_max_ and AUC_0–∞_ values for delta tocotrienol have been adjusted by dividing them with their respective dosage in the test and reference formulations.

For both gamma and delta tocotrienols, the 90% CI for both C_max_ and AUC_0–∞_ were beyond the bioequivalence limit of 80% to 125%. Hence, gamma and delta tocotrienols in both compounds were not bioequivalent. However, gamma tocotrienol in the test formulation was found to possess greater bioavailability in comparison to the gamma tocotrienol in the reference formulation. The significance in T_max_ values were analysed using Wilcoxon signed rank test. The T_max_ did not reach the significant statistical difference in both formulations for both gamma and delta tocotrienols.

### Safety and Tolerability

This study is the first to test GDT with self-emulsification system in humans. No adverse events were observed or reported in the study. All laboratory blood test results for all the subjects were within clinically acceptable range for both periods indicating the safety and tolerability of the drug.

## Discussion

In this study, we evaluated a newly developed GDT which only contained gamma and delta isomers of tocotrienol. This formulation was aimed to improve bioavailability of gamma and delta tocotrienols as these two isomers were reported to exert the most potent anti-cancer effects in several studies. These isomers increased the expression of Interleukin (IL)-24 mRNA which is associated with anti-tumour effects and decreased the levels of the pro-angiogenic cytokines, IL-6 and IL-8 in murine mammary cancer cells (4T1) and human umbilical vein endothelial cells (HUVEC)^22^,^23^. Apart from breast cancer, gamma tocotrienol has also been investigated in other types of cancer such as pancreatic^24^, hepatocellular^25^ and prostate cancer^26^ with favourable outcomes. Previous studies have reported that intake of tocotrienols with high fat meal increases its bioavailability as vitamin E is hydrophobic. Bile salts which will increase immensely after a high fat meal will emulsify the vitamin E and eventually form micelles that lead to increased bioavailability^20^,^27^. In this study, we have decided to administer the product after a normal standardized meal as both the formulations contained self-emulsifiers to aid in the absorption of tocotrienols across the intestinal wall. Furthermore it was a real reflection when the patient takes the product regularly with food in their daily life. Biphasic absorption pattern was observed in the plasma profile of some volunteers for both test and reference formulations. The secondary peak may be attributed from the redistribution of drug due to the involvement of the entero-hepatic system^20^.

Based on the ANOVA results for C_max_ and AUC_0–∞_, gamma tocotrienol in the test formulation (GDT) had superior bioavailability than the reference formulation (TRF). However, this was not observed for the delta tocotrienol which has slightly lower bioavailability for GDT as compared to TRF based on the ratio of the dose given. In a study carried out by Nesaretnam *et al*. (2007)^28^, the levels of different isomers of tocotrienols were analysed in adipose tissue of benign and malignant breast lumps of Malaysian women. Palm oil is the major oil consumed by these women in their daily dietary intake. Gamma tocotrienol was found in higher concentration in both benign and malignant tissue in contrast to delta tocotrienol. However, both gamma and delta tocotrienols concentrations were reduced in malignant breast lumps in comparison to the benign lumps. The morphology of the breast indicates that it is mainly composed of adipose tissue. Being a lipophilic compound with the presence of an unsaturated side chain, tocotrienols are able to penetrate efficiently into adipose tissue. The depletion of tocotrienols in malignant lumps might be associated with its antioxiodant property in quenching free radicals thereby suggesting its chemoprotective effect against breast cancer. Subsequently, Patel and colleagues (2012)^29^ looked at the levels of the isomers upon supplementation of TRF in surgical and normal patients. The concentration of gamma tocotrienol was found to be significantly higher in adipose tissue in comparison to delta tocotrienol. These findings further reinforces the notion that gamma tocotrienol plays a more prominent role in conferring protection against breast cancer.

Moreover, both formulations at the tested dose were found to be well tolerated in the healthy volunteers as no adverse events were observed and recorded during the entire period of the study.

The findings from our study demonstrate that the 600 mg GDT and 800 mg TRF, the nearly equivalent dose of gamma and delta tocotrienols were not bioequivalent in the healthy volunteers. GDT has shown superior bioavailability in term of gamma tocotrienol as compared to TRF. Both test and reference formulations appeared to be generally safe and well-tolerated in all the subjects.

## Additional Information

**How to cite this article**: Meganathan, P. *et al*. A new formulation of Gamma Delta Tocotrienol has superior bioavailability compared to existing Tocotrienol-Rich Fraction in healthy human subjects. *Sci. Rep*. **5**, 13550; doi: 10.1038/srep13550 (2015).

## Figures and Tables

**Table 1:** Demographic data for healthy volunteers.

Subject ID	Gender	Age	Height (cm)	Weight (kg)	BMI (kg/m^2^)
1	M	22	178	84	26.51
2	M	23	170	55	19.03
3	M	24	166	52	18.87
4	M	23	158	67	26.84
5	M	22	157	69	27.93
6	M	23	170	81.5	28.2
7	F	23	155	55.5	23.1
8	M	24	152	48	20.77
9	F	26	157	51	20.69
10	F	25	150	45	20.0
11	M	26	173	76	25.39
12	M	27	169	68	23.80
Mean ± SD		24 ± 1.65	162.92 ± 9.14	62.67 ± 13.36	23.43 ± 3.5
Minimum		22	150	45	18.87
Maximum		27	178	84	28.2

BMI = Body mass index.

M = Male.

F = Female.

**Table 2:** Mean pharmacokinetics parameters and bioequivalence results for Gamma Tocotrienol in 11 subjects after administration of GDT and TRF.

PARAMETERS	GDT3	TRF
T_max_ (h)	5.64 ± 1.50	4.73 ± 0.90
C_max_ (μg/L)	8406.75 ± 3670.99	5604.67 ± 1971.73
AUC_0–24_ (μg/L*h)	39811.07 ± 13336.74 μg/L*h	23312.73 ± 9804.14
AUC_0–∞_ (μg/L*h)	41091.37 ± 13406.89	24256.29 ± 10591.03
Kel (1/h)	0.28 ± 0.12	0.31 ± 0.20
Half-life (h)	2.97 ± 1.31	3.45 ± 2.50

**Table 3:** Mean pharmacokinetics parameters and bioequivalence results for Delta Tocotrienol in 11 subjects after administration of GDT and TRF.

PARAMETERS	GDT3	TRF
T_max_ (h)	5.18 ± 0.40	5.18 ± 1.83
C_max_ (μg/L)	2693.89 ± 962.7	2619.99 ± 1355.89
AUC_0–24_ (μg/L*h)	12136.42 ± 6417.29	10454.42 ± 6042.03
AUC_0–∞_ (μg/L*h)	14627.95 ± 9792.93	12453.83 ± 8875.65
Kel (1/h)	0.30 ± 0.28	0.38 ± 0.46
Half-life (h)	5.48 ± 4.75	5.11 ± 4.94

**Table 4:** ANOVA results for mean maximum concentration (C_max_) and area under curve (AUC) from the time 0 hours to infinity for Gamma Tocotrienol.

Parameters	Treatment	Mean (n = 11)	Ratio (%)	90% CI (%)	Bioequivalence
C_max_ (μg/L)	TRF	5604.67 ± 1971.73	144.08	106.21–195.46	NO
	GDT	8406.75 ± 3670.99			
AUC_0–∞_ (h*μg/L)	TRF	24256.29 ± 10591.03	173.77	154.11–195.93	NO
	GDT	41091.37 ± 13406.89			

Analysis Of Variance (ANOVA).

**Table 5:** ANOVA results for mean maximum concentration (C_max_) and area under curve (AUC) (dose adjusted) from the time 0 hours to infinity for Delta tocotrienol.

Parameters	Treatment	Mean (n = 11)	Ratio (%)	90% CI (%)	Bioequivalence
C_max_ (μg/mL/mg)	TRF	0.03 ± 0.01	72.23	52.35–99.66	NO
	GDT	0.02 ± 0.01			
AUC_0–∞_ (hr*μg/mL/mg)	TRF	0.10 ± 0.06	81.81	74.82–89.44	NO
	GDT	0.08 ± 0.04			
